# Experimental revival of an unknown state from the past in quantum walks

**DOI:** 10.1093/nsr/nwae263

**Published:** 2024-08-09

**Authors:** Bingzi Huo, Dengke Qu, Quan Lin, Gaoyan Zhu, Lei Xiao, Xiang Zhan, Peng Xue

**Affiliations:** Beijing Computational Science Research Center, Beijing 100193, China; Beijing Computational Science Research Center, Beijing 100193, China; School of Physics, Southeast University, Nanjing 211189, China; College of Electronic and Information Engineering, Shandong University of Science and Technology, Qingdao 266590, China; School of Physics, Southeast University, Nanjing 211189, China; School of Physics, Southeast University, Nanjing 211189, China; Beijing Computational Science Research Center, Beijing 100193, China

**Keywords:** quantum walk, quantum state revival, quantum state engineering

## Abstract

The physical process in the macroscopic world unfolds along a single time direction, while the evolution of a quantum system is reversible in principle. How to recover a quantum system to its past state is a complex issue of both fundamental and practical interests. In this article, we experimentally demonstrate a novel method for recovering the state in quantum walks (QWs), also known as full-state revival. Moreover, we observe two other important phenomena in QWs, recurrence and periodicity, via simplifying and repeatedly implementing the scheme, respectively. Our experiments show that obtaining these phenomena requires neither any information of the initial state nor full information of the coin operations. Our work sheds new light on quantum state engineering and recovery, and the initialization of quantum devices based on QWs.

## Teaser

A photonic walker is restored to its past state without requiring any information about the initial state or full details of the coin operations.

## INTRODUCTION

**Figure 1.**
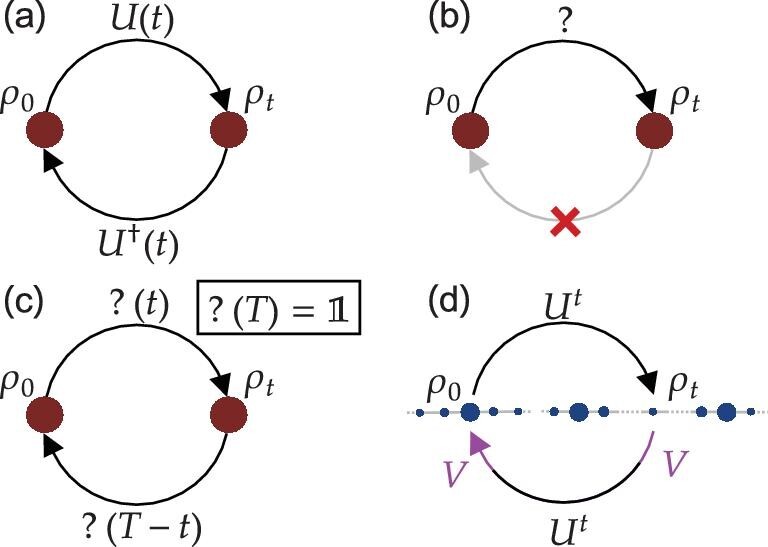
Illustration of the recovery of the initial state $\rho _0$ of a QW after the *t*-step evolution. (a) The evolved state $\rho _t$ can be recovered to $\rho _0$ by implementing the inversion evolution $U^\dagger (t)$ when knowing $U(t)$. (b) Recovering is impossible if one has no prior information about the previous evolution. (c) Knowing period *T* of the previous evolution enables the recovery of the state by letting it continue the evolution for a time ${\rm mod}\, (T-t,T)$. (d) FSR of an arbitrary evolved state of the QW induced by two intervention operations *V*.

Reversibility is an intrinsic feature of closed quantum systems, including ideal quantum computers, which is guaranteed by the unitarity of quantum evolution. Therefore, in principle, it is possible to recover a quantum system to its state at any past moment, as shown in Fig. 1(a), thus challenging the rule in the macroscopic world that physical processes unfold along a single time direction. Given a quantum system, the issue of recovering its state in the past is of fundamental and practical interests [1–4]. In general, this issue is impossible when no prior information is known about the evolution of the system. However, partial information, e.g. the periodicity of the evolution as shown in Fig. 1(c), might make the issue possible.

The studies of revivals and recurrences in quantum walks (QWs) are interesting since the behavior of QWs is ballistic spreading [5–10] and the QW has become a leading platform for both quantum computation [11–19] and quantum simulation [20–32]. Recovering states of the QW is always called a full-state revival (FSR) [33–42], which describes the walker-coin system returning exactly to the certain joint state. Similar to the FSR, recurrence [43–46] is another important phenomenon in the QW, which describes the probability distribution returning to the original distribution. Notably, the recurrence of the QW is an analogy of the recurrence in classical random walks, while the FSR has no classical counterpart [36]. It is obvious that if an FSR occurs, there must be a recurrence, but not vice versa. In previous literature [36–41,43,44], both FSRs and recurrences are called revivals and are always defined with a requirement of periodicity, which is another interesting phenomenon in QWs. However, the periodicity is sufficient but not necessary for an FSR in our definition. The relationship between these phenomena is


(1) \begin{eqnarray*}
{\text{Periodicity}} \subsetneq {\text{FSR}}\subsetneq {\text{Recurrence}} .
\end{eqnarray*}

Furthermore, it is worth noting an intriguing aspect: a protocol for FSR can be used to realize periodicity by periodically implementing the FSR protocol, which we demonstrate later.

The FSR has been widely observed as an attribute of the QW with periodicity due to a special architecture of the lattice, such as a QW on a circle [38,47]. However, for a QW on an infinite line, which is a basic model of the QW without general periodicity, observing the FSR requires complicated engineering of the QW with coin operations dependent on both the position and step [37]. Similar schemes have also been proposed for QWs on high-dimensional lattices [33,34,39].

In this article, we aim to introduce an FSR and periodicity of the QW on a line using limited information and controls. Motivated by the proposal of Jayakody *et al.* [33,34], we induce an FSR in a standard coined QW on a line by introducing intervention steps to replace the normal steps. With only two interventions on the coin system, the revivals of an arbitrary evolved walker-coin state are manifested after an arbitrary even number of steps. Compared to the previous proposal in [33,34], in which an FSR of the initial state of the system is implemented, we provide an improved method. As illustrated in Fig. 1(d), an arbitrary evolved state of a QW can be revived with only two interventions on the coin system introduced in any subsequent time step (see the online supplementary material-I). Furthermore, we demonstrate our method with a photonic QW platform, in which a photon walks on its orbital angular momentum (OAM) space under the control of its polarization state. The experimental results of the FSR are characterized by the quantum Pólya number and the state overlap.

## RESULTS AND DISCUSSION

### Proposal

In a QW on a line, the walker traces the evolute of a one-dimensional lattice $\mathbb {Z}$ controlled by its two-dimensional coin state. The Hilbert space of the joint system is thus spanned by the basis $\lbrace |{x,z}\rangle :x\in \mathbb {Z},\, z=\pm 1\rbrace$. The evolution of each step is $U=SC$, where the conditional shift operator acts as $S|{x,z}\rangle =|{x\pm (-1)^z,z}\rangle$ and the coin-flipping operator *C* acts solely on the coin state. Here we consider a homogeneous coin-flipping operation [48]


(2) \begin{eqnarray*}
C=\mathbb {1}_{w}\otimes \left(\begin{array}{c@{\quad\,\, }c}\cos \theta & e^{i\phi _{1}}\sin \theta \\
e^{i\phi _{2}}\sin \theta &e^{i(\phi _{1}+\phi _{2})}\cos \theta \end{array}\right),
\end{eqnarray*}

where $\mathbb {1}_{w}$ denotes an identity operator in the position space. Here parameters $\phi _1$ and $\phi _2$ are phase factors, and $\theta$ denotes the bias of coin flipping that determines the ballistic behavior of the QW. In the static case when the coin operations are independent of steps, the state of the system after *t* steps is $|\psi _{t}\rangle =U^{t}|\psi _{0}\rangle$, where $|{\psi _0}\rangle$ is the initial state.

To fully revive an evolved state of the QW with limited control, another homogeneous coin-flipping operation is introduced [33,34]:


(3) \begin{eqnarray*}
G=\mathbb {1}_{w}\otimes {\left(\begin{array}{cc}0 & e^{i\phi _{1}} \\
-e^{i\phi _{2}} &0 \end{array}\right)}.
\end{eqnarray*}

This coin operation *G* satisfies the relations [33,34]


(4) \begin{eqnarray*}
&&GG=-e^{i\Phi }\mathbb {1}, \qquad G^\dagger C G^\dagger =C^\dagger , \\
&& GSG^\dagger =G^\dagger SG=S^\dagger ,
\end{eqnarray*}

where $\Phi =\phi _1+\phi _2$. For a certain step of the QW, we replace the coin operation with *G* and the evolution is then $V=SG$. The certain step is called an intervention step. An FSR of an arbitrary evolved state of the QW is induced by two intervention steps introduced in arbitrary subsequent time steps. For example, the first intervention step is introduced at step *T*; the state for step $t\ge T$ is then


(5) \begin{eqnarray*}
|{\psi _T}\rangle =VU^{T-1}|{\psi _0}\rangle .
\end{eqnarray*}

After another intervention step at step $t\in [T+1,\dots ,2T]$, the final state becomes


(6) \begin{eqnarray*}
|{\psi _{t}}\rangle &=&V U^{t-T-1}|{\psi _T}\rangle\\
&=&{(-1)^{t-T}}e^{(t-T)i \Phi }U^{2T-t} |{\psi _0}\rangle \propto |{\psi _{2T-t}}\rangle. \\
\end{eqnarray*}

An FSR of an arbitrary evolved state $|{\psi _{2T-t}}\rangle$ is achieved by properly choosing the second intervention step *t*. If $t=2T$, the FSR of the initial walker-coin state is achieved. Introducing periodicity to the QW is straightforward by periodically implementing the intervention step. If the intervention operation is implemented every *T* steps, the period of the QW is $2T$.

Furthermore, we find that the recurrence happens two steps earlier than the FSR. This is because the last two steps with operations $VU$ do not change the distribution of the walker in position space as $VU=SGSC=GG^\dagger SGSC=GS^\dagger S C=GC$. Therefore, obtaining the recurrence of any past distributions with only one intervention operation is much simpler.

### Experimental setup

**Figure 2.**
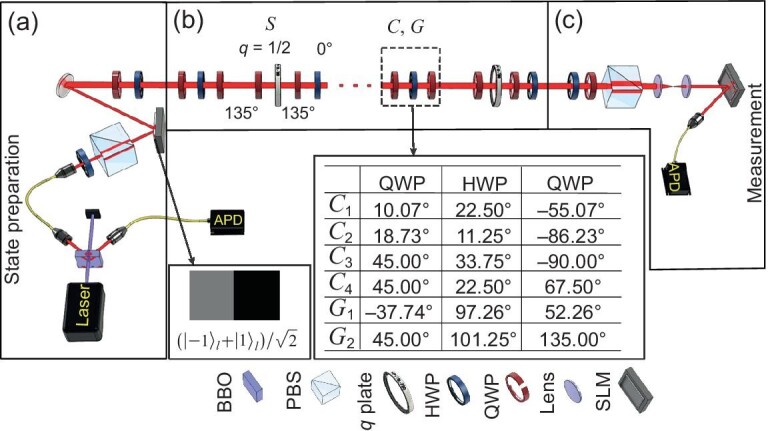
Experimental setup. (a) State preparation. Herald single photons are generated and initialized in a horizontal-polarized Hermite-Gaussian mode. The inset shows the phase hologram for preparing the superposed position state. (b) QW realized by wave plates and *q* plates. The inset shows the setting angles of wave plates for different coin operations. Here $C_{1}{:}\ \phi _{1} =\phi _{2} =0, \theta =\pi /4; C_{2}{:}\ \phi _{1} =\phi _{2} =0, \theta =\pi /8; C_{3}{:}\ \phi _{1} =0, \phi _{2} =\pi /4,\theta =\pi /4; C_{4}{:}\ \phi _{1} =0,\phi _{2} =\pi /4,\theta =\pi /8$ and $G_{1}{:}\ \phi _{1} =\phi _{2} =0; G_{2}{:}\ \phi _{1} =0, \phi _{2} =\pi /4$. (c) Measurement is realized by wave plates, a polarizing beam splitter (PBS), a spatial light modulator (SLM) and an avalanche photodiode.

We experimentally demonstrate our scheme for revival with a photonic QW platform. Photon pairs at a wavelength of 780 nm are generated via a type-I spontaneous parametric down-conversion process in pumping a $\beta$-barium-borate (BBO) nonlinear crystal with light at a wavelength of 390 nm. As shown in Fig. 2, the idler photon is detected by an avalanche photodiode (APD) to herald the other signal photon, which is injected into the QW platform to act as the walker.

In the QW platform, the coin’s and walker’s positions are encoded in the polarization ($|H\rangle$ and $|V\rangle$) and OAM ($|{l}\rangle ,\, l\in \mathbb {Z}$) modes of the heralded single photons, respectively. Here OAM is the internal mode of the photon related to the non-trivial transverse space wave front [49–51], which has been wildly used for high-dimensional quantum information processing [52–61]. It is worth mentioning that both the OAM mode and the position space of the QW have infinite dimensions. However, the QW with an initial state distributed in finite positions evolves on finite positions. Therefore, we implement the QW in finite positions using finite OAM modes.

We generate a TEM$_{00}$ mode by coupling heralded single photons to a single-mode fiber (SMF), which initializes the beam in an OAM state with $l=0$. After that, horizontal polarization is chosen by a polarization beam splitter (PBS). That is, the photons are initially in a Hermite-Gaussian (HG) mode with horizontal polarization. To generate different initial states, a spatial light modulator (SLM) and wave plates (WPs) are used to manipulate the OAM and polarization modes of the photons, respectively. The SLM maintains the OAM modes of vertically polarized photons unchanged and converts the HG mode of the horizontally polarized photons into different Laguerre-Gaussian modes corresponding to the loaded phase hologram. In this article, we consider three different initial states: $| \psi ^{1} _{0} \rangle = |0 \rangle \otimes ( | H \rangle +i | V \rangle ) /\sqrt{2}$, $| \psi ^{2} _{0} \rangle = |0 \rangle \otimes | H \rangle$ and $| \psi ^{3} _{0} \rangle =( |1 \rangle + |-\!1 \rangle ) /\sqrt{2} \otimes ( | H \rangle +i | V \rangle )/\sqrt{2}$.

Here the walker state $(|{1}\rangle +|{-1}\rangle )/\sqrt{2}$ is generated by the SLM with phase hologram shown in Fig. 2(a), and the coin state, i.e. the specific polarization $(|{H}\rangle +i|{V}\rangle )/\sqrt{2}$, is selected by a quarter-wave plate (QWP) with a setting angle of $135^\circ$.

The conditional shift operator is realized by a QWP–*q*-plate–QWP–HWP array, as shown in Fig. 2(b), where the setting angles of the three WPs are $135^\circ$, $135^\circ$ and $0^\circ$. Here, a *q* plate is an optical device formed by a thin layer of liquid crystals [60,62] with a singular pattern of optic axes characterized by a topological charge *q*. In our experiment, the topological charge is $q=1/2$ to ensure that the change in the OAM state is $2q=1$ [57]. The structured birefringence leads to an optical spin-orbit coupling, causing a polarization-dependent shift in the OAM as


(7) \begin{eqnarray*}
\mathrm{S}_{q}&=&\sum _{x} | x+2q \rangle \langle x| \otimes | R \rangle \langle L|\\
&& +\, | x-2q \rangle \langle x| \otimes | L \rangle \langle R|,
\end{eqnarray*}

where $|{L}\rangle =(|{H}\rangle +i|{V}\rangle )/\sqrt{2}$ and $|{R}\rangle =(|{H}\rangle -i|{V}\rangle )/\sqrt{2}$.

The homogeneous coin operations are realized by an array of WPs. In this article, we consider four kinds of coin-flipping operations, of which the setting angles of the WPs are shown in Fig. 2(b).

The final state of the QW is projected by a combination of WPs followed by a PBS. The walker state can be measured by diffraction on an SLM. As shown in Fig. 2(c), we first project the coin state into $|{H}\rangle$ and then use an SLM to transform the walker state into an HG mode, which is then selected by an SMF. The photon is finally detected by APDs in coincidence with the detection of a trigger photon with a time window of 3 ns. For each measurement, we record the photon counts for 3 s and the total coincidence count is about 7500.

In our experiment, the error sources include the efficiencies of the *q* plates, the spatial light modulator and the avalanche photodiodes, respectively, and the accuracy of the wave plates. Among these, the efficiency of the *q* plate (approximately 0.995) is the main source of experimental imperfections (see the SM-VI).

### Experimental results

**Figure 3.**
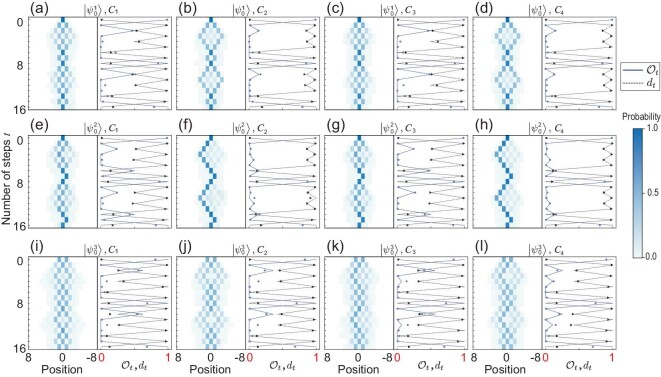
Experimental results of the QW with three different initial states and four different coin parameters. The first intervention is introduced at ${T=4}$. In each panel, the left plot is the measured probability distribution and the right shows the experimental results of TV distances $d_t$ (triangles) and overlaps $\mathcal {O}_t$ (circles). Symbols represent the experimental data and lines are the corresponding theoretical predictions of $d_t$ and $\mathcal {O}_t$. Error bars are due to the statistical uncertainty in photon-number counting and some of them are smaller than the symbol size. Each row employs the same initial state as $|{\psi _{0}^{1}}\rangle$ for (a)–(d), $|{\psi _{0}^{2}}\rangle$ for (e)–(h) and $|{\psi _{0}^{3}}\rangle$ for (i)–(l). Each column employs the same set of coin-flipping operators as $C_{1}$ for (a), (e), (i); $C_{2}$ for (b), (f), (j); $C_{3}$ for (c), (g), (k) and $C_{4}$ for (d), (h), (l).

An important quantity of the QW is the position distribution of the walker. The probability of finding the walker at position *x* at step *t* is $P_{t}^{x}=\sum _z\langle {\psi _t}|x\rangle \langle x|\otimes |z\rangle \langle z|{\psi _t}\rangle$. Experimentally, it can be calculated by the measured photon numbers, i.e. $\bar{P}_{t}^{x}=(\sum _{z}N_{t,x,z})/(\sum _{x^\prime ,z}N_{t,x^\prime ,z})$, where $N_{t,x,z}$ is the number of photons when measuring the system at step *t* with projective measurement $|{x}\rangle \!\langle {x}|\otimes |{z}\rangle \!\langle {z}|$. The probability distributions of our experiments are shown in Fig. 3 for $T=4$ and in the SM-IV for $T=5$. We quantify the experimental demonstration of the QW via the similarity $S_t= {\sum _{x}} \sqrt{P_t^x \bar{P}_{t}^x}$ [59], ranging from 0 to 1 for completely orthogonal and identical distributions, respectively. The average similarities of the total 12 final states of the $2T$-step QWs with three different initial states and four different coin parameters are $0.9322\pm 0.0048$ for $T=4$ and $0.9167\pm 0.0061$ for $T=5$, and those of the $4T$-step QWs are $0.9179\pm 0.0049$ and $0.9145\pm 0.0081$, respectively, which show high reliability of our experiment (see the SM-IV for details).

Recurrence denotes that the position distribution of the walker returns to a previous distribution. The difference between the two distributions is characterized by a total-variation (TV) distance [37,63]


**Figure 4.**
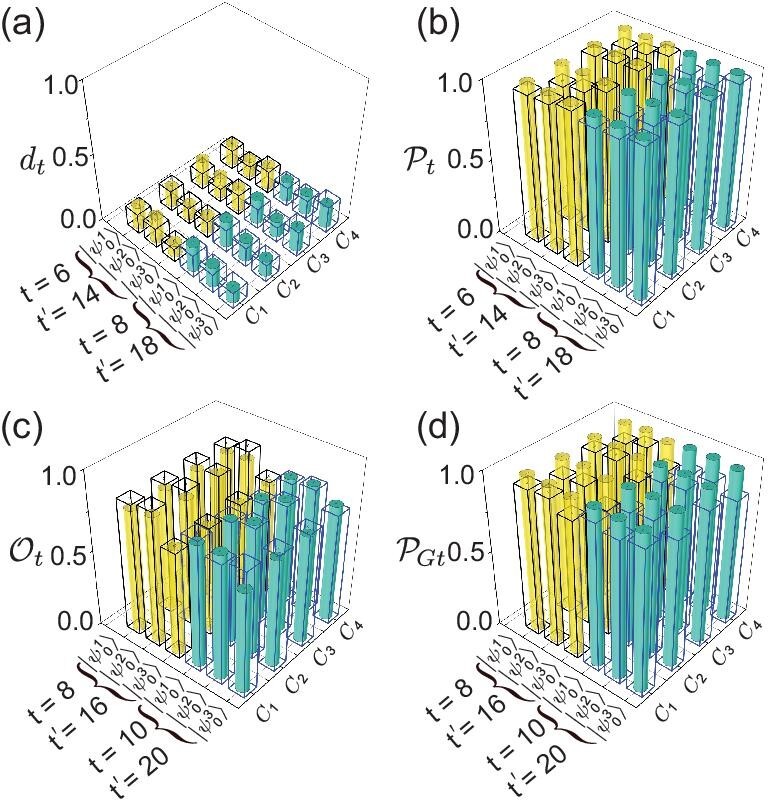
Experimental results of the QW with three different initial states and four different coin parameters. We consider two cases in which the first intervention is introduced at $T=4$ (yellow) and $T=5$ (blue), respectively. (a) TV distances $d_t$ and (b) QW Pólya numbers $\mathcal {P}_t$ for $t=2T-2$ and $t^{\prime }=4T-2$. (c) Overlaps $\mathcal {O}_t$ and (d) generalized Pólya numbers ${\mathcal {P}_{G}}_t$ for $t=2T$ and $t^{\prime }=4T$. Solid and hollow bars represent the experimental results for $t^{\prime }$ and *t*, respectively.

(8) \begin{eqnarray*}
d_{t}=\frac{1}{2}\sum _{x} \left| \bar{P}_t^x-P_{0}^x \right|,
\end{eqnarray*}

which ranges from 0 for two identical distributions to 1 for orthogonal distributions. As shown in Fig. 4(a), the average TV distances of the total 12 final states of the $(2T-2)$-step QWs with three different initial states and four different coin parameters are $0.1427\pm 0.0066$ for $T=4$ and $0.1502\pm 0.0046$ for $T=5$. The average TV distances of the $2T$-step QWs are $0.1575\pm 0.0050$ for $T=4$ and $0.1633\pm 0.0074$ for $T=5$. Hence, the recurrence of the QW is achieved at the time steps $2T-2$ and $2T$.

Under the condition that the walker distribution is localized, the distance is simply related to the QW Pólya number [43,44,64–66]


(9) \begin{eqnarray*}
\mathcal {P}_t=1-{\prod _{t^\prime =1}^t} d_{t^\prime },
\end{eqnarray*}

which is a quantum version of Pólya number in classical random walks. A QW is recurrent only if the QW Pólya number is 1. As shown in Fig. 4(b), the experimental results of the quantum Pólya number of the total 12 final states of the $(2T-2)$-step QWs with three different initial states and four different coin parameters are $0.9520\pm 0.0006$ for $T=4$ and $0.9515\pm 0.0014$ for $T=5$, which agree well with their theoretical predictions of 1.

Beyond recurrence, an FSR requires the state of the QW to return to a certain initial state. We use the state overlap as a figure of merit, i.e. $\mathcal {O}=\langle {\psi _0}|\psi _t\rangle \langle \psi _t|{\psi _0}\rangle$. In principle, to obtain the overlap, a full state tomography of the initial and final states is needed, which is challenging, especially for the case with a large number of steps [67–69]. However, the final state is compared to the initial state, so we only perform quantum state tomography in the positions occupied by the initial state. The overlap can be rewritten as $\mathcal {O}_t^{\prime }=P^{\prime }_t \text{Tr}(\rho _{t}|{\psi _0}\rangle \langle {\psi _0}|)$, where $\rho _t$ is the density matrix of the state on these special positions and $P^{\prime }_t$ is the probability of finding the walker at these positions. As shown in Fig. 3, the average overlaps $\mathcal {O}_{2T}$ are $0.7674\pm 0.0081$ for $T=4$ and $0.7738\pm 0.0049$ for $T=5$, which certifies the FSR at step $2T$ as predicted.

Similar to recurrence, one can define a generalized Pólya number to quantify revivals, which can be obtained through overlaps as [33]


(10) \begin{eqnarray*}
{\mathcal {P}_G}_t=1-{\prod _{t^\prime =1}^t} (1-\mathcal {O}_{t^\prime }).
\end{eqnarray*}

There is an FSR in a *t*-step QW if and only if the generalized Pólya number ${\mathcal {P}_G}_t$ is 1. It is to be noted that the definition of ${\mathcal {P}_G}_t$ is similar to $\mathcal {P}_t$ with the key distinction lying in its comparison of quantum states as opposed to the classical probability distributions compared by the latter. The generalized Pólya numbers of a $2T$-step QW are shown in Fig. 4(d). The average Pólya numbers $\mathcal {P}_{Gt}$ of the $2T$-step QWs with three different initial states and four different coin parameters are $0.8948\pm 0.0043$ for $T=4$ and $0.8902\pm 0.0044$ for $T=5$, which agree well with their theoretical predictions of 1.

Since the method revives the initial state after step $2T$, the dynamics of the following steps should be identical to these $2T$ steps. Therefore, periodicity, when applying the method for revival each $2T$ steps, would introduce a periodicity to the QW with a period of $2T$. We continue the QW to step $4T$ for each QW to observe the periodicity. As shown in Figs 3 and 4 and the SM-VI, the experimental results exhibit recurrence, and the FSR appears again at steps $4T-2$ and $4T$.

It is worth mentioning that our scheme for FSR can revive the state of an arbitrary previous state. We have implemented an experiment to revive state $|{\psi _{2}^{2}}\rangle $ that has been evolved for $t=2$ steps. After implementing an intervention operation at steps $t=4$ and $t=6$, the state is revived at step $t=6$, i.e. $|{\psi _{6}^{2}}\rangle =|{\psi _{2}^{2}}\rangle $ (see the SM-V for details).

## CONCLUSIONS

In summary, we have proposed and experimentally demonstrated a method for the FSR in QWs on a line. This method can be simplified to obtain the loose phenomenon of recurrence and can also be repeatedly used to achieve periodicity. A critical feature of this method is that it does not depend on either the initial state or the coin flipping. An arbitrary evolved state of a QW can be revived with only two interventions on the coin system introduced in any subsequent time step without the requirement of any prior information of the state. This brings new ideas to the important issue of state recovery [1–4]. Our work sheds new light on engineering QWs and is expected to provide an initialization method for quantum information processors based on QWs.

## Supplementary Material

nwae263_Supplemental_File
