# Supplementary material for: Experimental revival of an unknown state from the past in quantum walks
Source: Natl Sci Rev. 2024 Aug 9;12(1):nwae263. doi: 10.1093/nsr/nwae263 (PMC11702667; doi:10.1093/nsr/nwae263)
Supplement: nwae263_Supplemental_File [file nwae263_supplemental_file.pdf]

Supplemental Material for "Experimental Revival of Unknown State From the Past in Quantum Walks"

I. COMPARISON BETWEEN OUR PROPOSAL AND THE PREVIOUS ONES

In Ref. [1], the authors model a scheme of quantum walks in which interventions on the coin space V are introduced at specific time steps as

$$|\psi_t\rangle = \cdots [U^{(l-1)}VU^l][U^{(l-1)}VU^l]\,|\psi_0\rangle \propto |\psi_0\rangle\,, \tag{1}$$

which represents a periodic quantum motion. It is equivalent to the event of introducing the operator V with $2l-1$ time gaps. Thus, for a full state revival of the initial state, they need at least $4l$ steps. However, in our proposal, we have

$$|\psi_t\rangle = \cdots VU^lVU^l\,|\psi_0\rangle\,. \tag{2}$$

Thus, for a full state revival of the initial state, we only need $2(l+1)$ steps. Moreover, we can backpedal the quantum walk not only to its initial state but also to any evolved state $|\psi_{t'}\rangle = U^{t'}|\psi_0\rangle$.

In Ref. [2], the authors model a scheme of quantum walks in which interventions on the coin space V are introduced at specific time steps as

$$|\psi_t\rangle = W_l^r\,|\psi_0\rangle\,, \tag{3}$$

where $W_l = U^lV$ and $t = r(l+1)$. They prove that

$$W_l^2 = e^{i\phi}I, \tag{4}$$

where ϕ is a global phase and I is an identity operator. Thus, they can backpedal the quantum walk to its initial state by applying W_l twice during a single cycle. In our proposal, we can backpedal the quantum walk not only to its initial state but also to any evolved state $|\psi_{t'}\rangle = U^{t'}|\psi_0\rangle$. As shown in Eqs. (5) and (6) of the main text, the first intervention step is introduced at the step T, i.e., we replace U by V at the step T, and then we have

$$|\psi_T\rangle = VU^{T-1}\,|\psi_0\rangle\,. \tag{5}$$

After another intervention V introduced at the step t, the finial state becomes

$$|\psi_t\rangle = VU^{t-T-1}\,|\psi_T\rangle \propto |\psi_{t'}\rangle\,, \tag{6}$$

where $t' = 2T - t$. Thus, an fully state revival of $|\psi_{t'}\rangle$ is achieved by properly choosing the second intervention step t. Compared to the previous theoretical proposal, we can backpedal the quantum walk not only to its initial state $|\psi_0\rangle$ but also to any evolved state $|\psi_{t'}\rangle$.

II. CONDITIONAL SHIFT OPERATOR

In our experiment, the conditional shift operator is implemented by two QWPs, one q-plate with the topological charge $q = 1/2$ and one HWP. The action of the q-plate can be generally described by the operator

$$\mathrm{S}_q = \sum_x |x+2q\rangle\langle x| \otimes |R\rangle\langle L| + |x-2q\rangle\langle x| \otimes |L\rangle\langle R|\,, \tag{7}$$

where $|L\rangle = (|H\rangle + i\,|V\rangle)/\sqrt{2}$ and $|R\rangle = (|H\rangle - i\,|V\rangle)/\sqrt{2}$. The q-plate shifts the OAM state of the beam by the value of $2q$ or $-2q$ depending upon whether the polarization state of the beam is $|L\rangle$ or $|R\rangle$. The action of the QWP $R_Q(\alpha)$ is addressed as

$$R_Q(\alpha) = \begin{pmatrix} (\cos\alpha)^2 + i(\sin\alpha)^2 & \frac{1-i}{2}\sin 2\alpha \\ \frac{1-i}{2}\sin 2\alpha & (\sin\alpha)^2 + i(\cos\alpha)^2 \end{pmatrix}. \tag{8}$$

The action of the HWP $R_H(\alpha)$ is addressed as

$$R_H(\alpha) = \begin{pmatrix} \cos 2\alpha & \sin 2\alpha \\ \sin 2\alpha & -\cos 2\alpha \end{pmatrix}. \tag{9}$$

In our experiment, the angles of the three WPs are 135°, 135°, and 0°, and the operator can be described as

$$S = R_H(0) \cdot R_Q(\frac{3\pi}{4}) \cdot S_Q^{q=\frac{1}{2}} \cdot R_Q(\frac{3\pi}{4}) = \sum_x |x+1\rangle \langle x| \otimes |H\rangle \langle H| + |x-1\rangle \langle x| \otimes |V\rangle \langle V| \tag{10}$$

III. EXPERIMENTAL DETAILS

TABLE I. Setting angle of WPs for project measurement of coin state.

Basis	QWP	HWP
$\|0\rangle_c$	0°	0°
$\|1\rangle_c$	0°	45.00°
$(\|0\rangle_c - i\|1\rangle_c)/\sqrt{2}$	0°	22.50°
$(\|0\rangle_c + \|1\rangle_c)/\sqrt{2}$	45.00°	22.50°

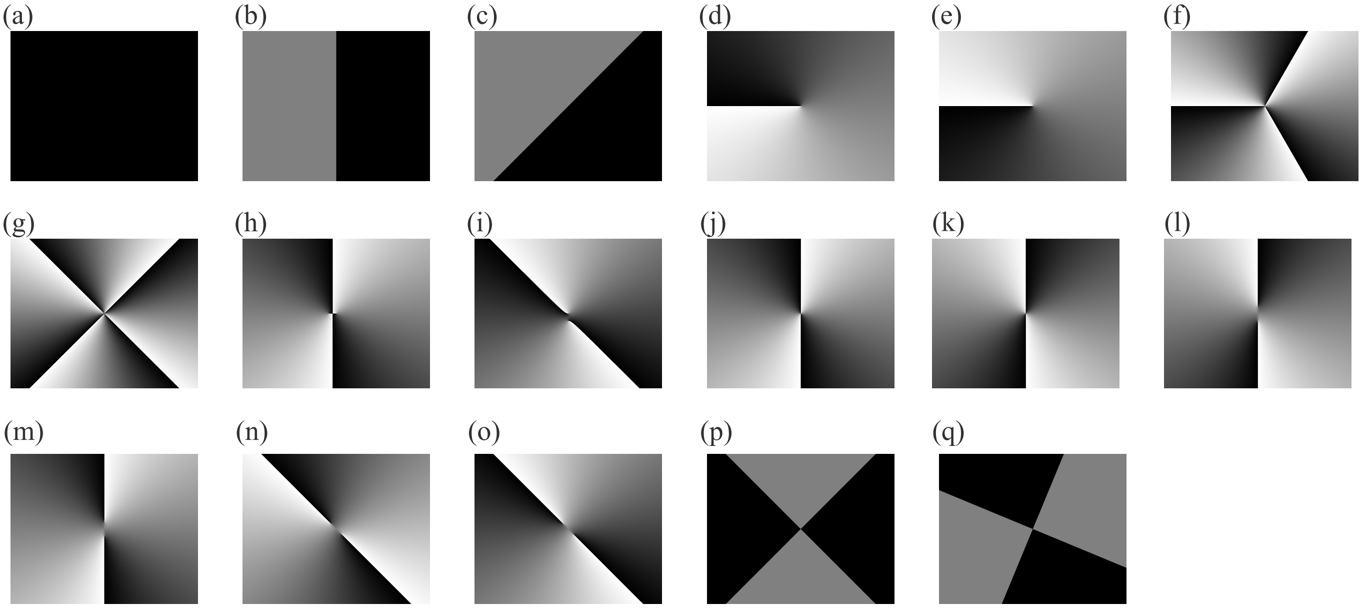

FIG. S1. Phase holograms loaded by the SLM for projective measurements of position state on basis of the initial states $|\psi_0^1\rangle$ and $|\psi_0^2\rangle$ in (a), $|\psi_0^3\rangle$ in (b-e), $|\psi_0^4\rangle$ in (f), $|\psi_0^5\rangle$ in (g), $|\psi_0^6\rangle$ in (e, h-j), $|\psi_2^2\rangle$ in (a, j-q), where (a) $|0\rangle_w$, (b) $(|1\rangle_w + |-1\rangle_w)/\sqrt{2}$, (c) $(|1\rangle_w - i|-1\rangle_w)/\sqrt{2}$, (d) $|-1\rangle_w$, (e) $|1\rangle_w$, (f) $|3\rangle_w$, (g) $|-4\rangle_w$, (h) $(|1\rangle_w + |2\rangle_w)/\sqrt{2}$, (i) $(|1\rangle_w - i|2\rangle_w)/\sqrt{2}$, (j) $|2\rangle_w$, (k) $|-2\rangle_w$, (l) $(|0\rangle_w + |-2\rangle_w)/\sqrt{2}$, (m) $(|0\rangle_w + |2\rangle_w)/\sqrt{2}$, (n) $(|0\rangle_w + i|-2\rangle_w)/\sqrt{2}$, (o) $(|0\rangle_w - i|2\rangle_w)/\sqrt{2}$, (p) $(|-2\rangle_w + |2\rangle_w)/\sqrt{2}$, (q) $(|-2\rangle_w + i|2\rangle_w)/\sqrt{2}$.

In the state preparation, we consider two coin states and two position states. The position state is initialized as $|0\rangle$. The phase hologram loaded by the spatial light modulator (SLM) to prepare position states $(|1\rangle + |-1\rangle)/\sqrt{2}$ are shown in Fig. 2 of the main text. The coin state is initialized as $|0\rangle$. Preparation of coin state includes a half-wave plate (HWP) followed by a quarter-wave plate (QWP). To prepare the coin states $|0\rangle$ or $(|0\rangle + i|1\rangle)/\sqrt{2}$, setting angles of the QWP and HWP are 0° and 0° or 45.00° and 0°, respectively.

The projective measurements of coin (position) state are realized by WPs (SLM). For the cases with initial states $|\psi_0^1\rangle$ or $|\psi_0^2\rangle$, we implement measurements on the subspace spanned by $|0\rangle_w |0\rangle_c$ and $|0\rangle_0 |1\rangle_c$. For the cases with initial states $|\psi_0^4\rangle$ ($|\psi_0^5\rangle$), we implement measurements on the subspace spanned by $|3\rangle_w |0\rangle_c$ and $|3\rangle_0 |1\rangle_c$ ($|-4\rangle_w |0\rangle_c$

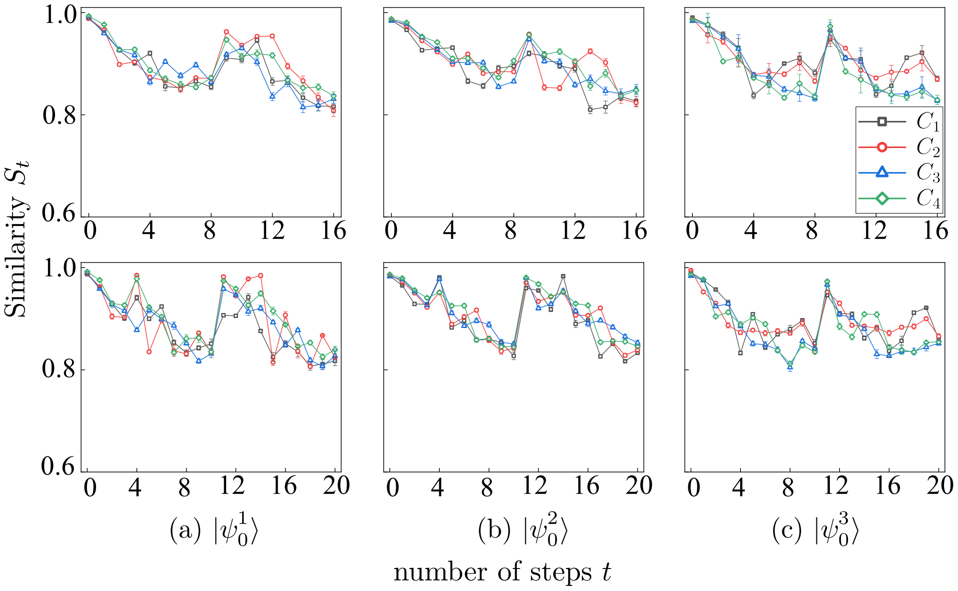

FIG. S2. Experimental data on probability similarity. (a)-(c) Initial state is $|\psi_0^1\rangle$, $|\psi_0^2\rangle$, $|\psi_0^3\rangle$ respectively. The first(second) line represents $2T = 4(2T = 5)$. The gray box,red circle,blue triangle, green diamond, indicates that the QW platform parameter is C_1, C_2, C_3, C_4 respectively.

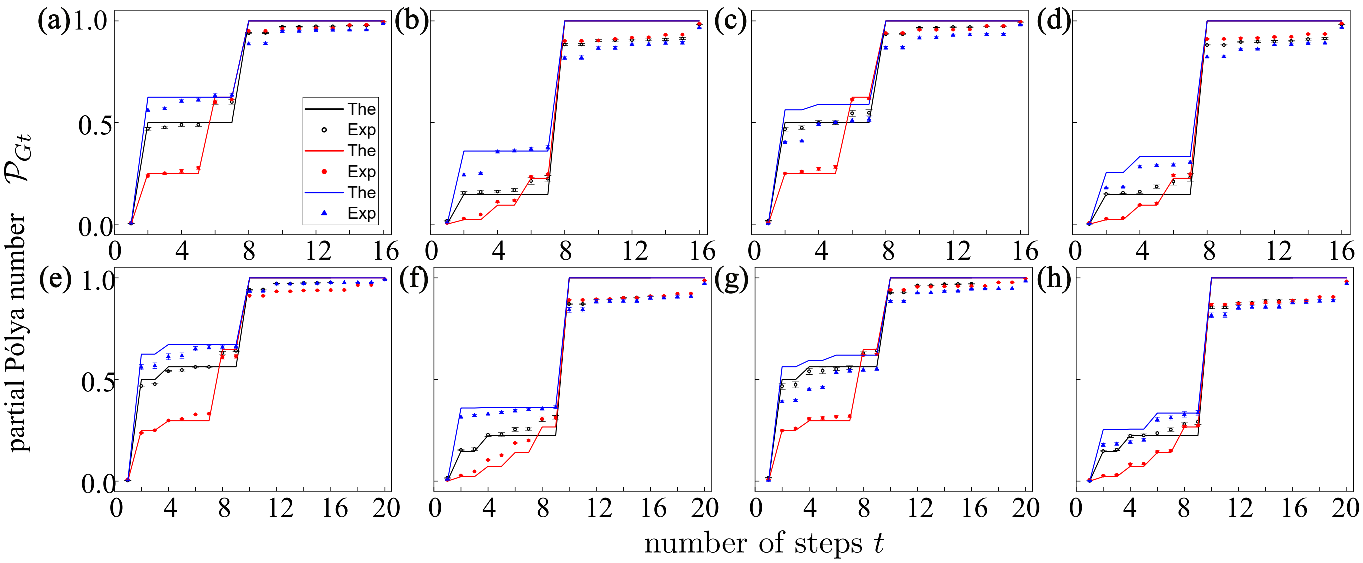

FIG. S3. Partial *Pólya* number of QWs. The solid line represents the theoretically calculated value. Black indicates that the initial state is $|\psi_0^1\rangle$, and the hollow dot is its experimental result. Red indicates that the initial state is $|\psi_0^2\rangle$, and the solid dot is its experimental result. The blue triangle is the result of $|\psi_0^3\rangle$. (a)-(d) with the period $2T = 8$ and (e)-(f) with the period $2T = 10$.

and $|-4\rangle_0 |1\rangle_c$). We obtained state $\rho_t^{1(2,4,5)}$ through quantum state tomography as shown in Table I. For the case with initial state $|\psi_0^3\rangle$ ($|\psi_0^6\rangle$), we implement measurements on the subspace spanned by $|1\rangle_w |0\rangle_c$, $|1\rangle_w |1\rangle_c$, $|-1\rangle_w |0\rangle_c$, and $|-1\rangle_w |1\rangle_c$ ($|1\rangle_w |0\rangle_c$, $|1\rangle_w |1\rangle_c$, $|2\rangle_w |0\rangle_c$, and $|2\rangle_w |1\rangle_c$. In total, we consider four projective measurements of coin states, shown Table I and four projective measurements of position states, shown in Figs. S1 (b-e) (Figs. S1(e, h-j)). Then, the final states are reconstructed through quantum state tomography, with 16 sets of projective measurements. For measurements of coin, the pairs of setting angles of the QWP and HWP are shown Table I . Phase holograms for the SLM to measuring the position state are shown in Figs. S1(c-f) (Figs. S1(e, h-j)).

For states in the process of evolution $|\psi_2^2\rangle$, we implement measurements on the subspace spanned by $|2\rangle_w |0\rangle_c$, $|2\rangle_w |1\rangle_c$, $|0\rangle_w |0\rangle_c$, $|0\rangle_w |1\rangle_c$, $|-2\rangle_w |0\rangle_c$ and $|-2\rangle_w |1\rangle_c$. In total, we consider four projective measurements of coin

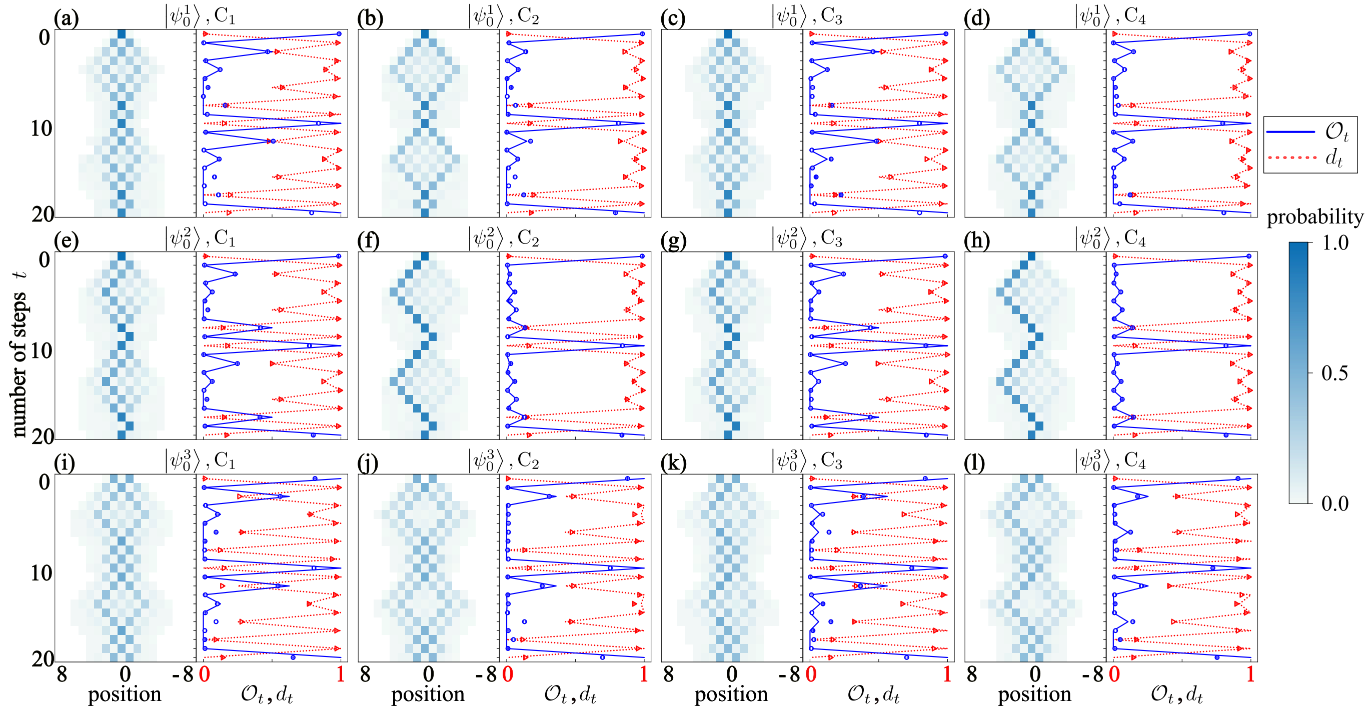

FIG. S4. Experimental results for period $T = 5$. In each subgraph, the left figure is the measured probability distributions and the right shows the experimental result of TV distances d_t (red triangle) and overlaps $\mathcal{O}_t$ (blue circle). Theoretical predictions of d_t and $\mathcal{O}_t$ are represented by red dotted and blue solid lines, respectively. Each column (row) represents the results with the same coin operation (initial state). Error bars are smaller than the symbol size.

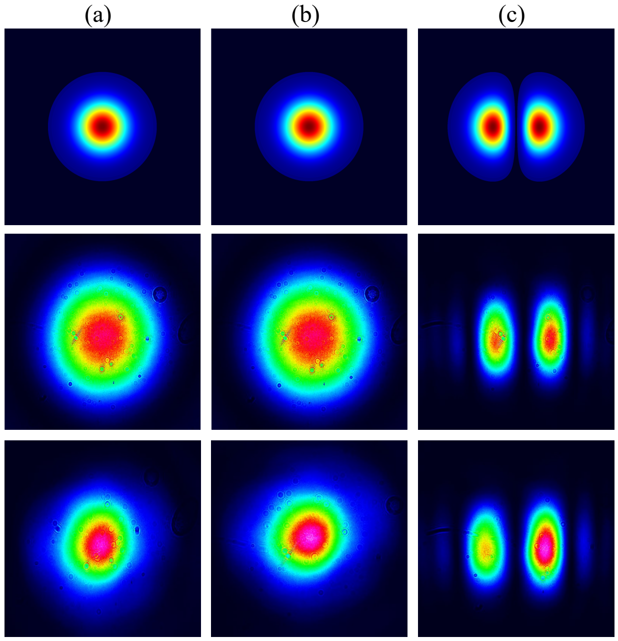

FIG. S5. Intensity distribution of OAM states obtained via numerical simulation (first row) and experiments (second and third row). The second and third rows represent the result at $t = 0$ and $t = 8$, respectively. Each experimental result is compared to the numerical case using similarity. Three columns correspond to the cases with three different initial states $|\psi_0^1\rangle$, $|\psi_0^2\rangle$ and $|\psi_0^3\rangle$, respectively.

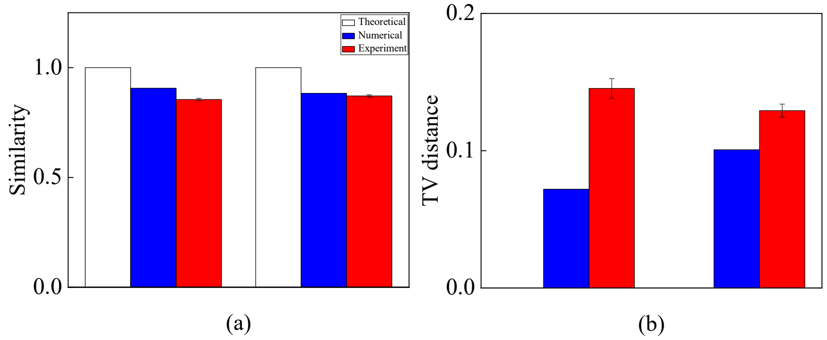

FIG. S6. Similarities (a) and TV distances (b) obtained via noiseless theoretical prediction, numerical simulation considering the conversion efficiency of q-plate, and experiment. For both quantities, we consider its value at the 8-th step corresponding the cases in Fig. 3 (a) and (b) in the main text. In these cases, the theoretical value of TV distances are 0. Error bars are due to the statistical uncertainty in photon-number-counting.

states, shown Table I and nine projective measurements of position states, shown in Figs. S1 (a, j-q). Then, the final states are reconstructed through quantum state tomography, with 36 sets of projective measurements.

IV. EXPERIMENTAL RESULTS

To quantify the reliability of our experiments, we calculate the similarity between experimental distributions and the theoretical prediction. The similarities in our experiments are shown in Fig. S2. We compare the reconstructed final state ρ_{exp} with its theoretical prediction ρ_{the} via the fidelity

$$F = \text{Tr}\sqrt{\sqrt{\rho_{\text{exp}}} \cdot \rho_{\text{the}} \cdot \sqrt{\rho_{\text{exp}}}}^{\,2}. \tag{11}$$

Here, the final states are reconstructed via quantum state tomography. For $T = 4$, the average fidelities at the end of two periods are $F_{2T} = 0.8207 \pm 0.0014$, $F_{4T} = 0.7819 \pm 0.0024$. For $T = 5$, the average fidelities are $F_{2T} = 0.8048 \pm 0.0018$, and $F_{4T} = 0.7827 \pm 0.0018$.

As described in the main text, revival can be quantified by the generalized Pólya number, which can be obtained through overlaps as

$$\mathcal{P}_{Gt} = 1 - \prod_{t'=1}^{t}(1 - \mathcal{O}_{t'}). \tag{12}$$

Results of Pólya numbers are shown in the Fig. S3, which certifies the obtaining of FSR within experimental errors.

In Fig. 3 of the main text, we show the experimental results of different initial states for $T = 4$. Our method is flexible for FSR of arbitrary time. To demonstrate this, we have implemented experiment with $T = 5$, of which the detailed results are shown in Fig. S4. Here we conduct quantum state tomography to estimate the state during the initial revival, followed by a re-preparation of the state for the subsequent period. According to these results, it is easy to see that FSR and peoriodicity can still be achieved.

The experimental error bar was obtained by Monte Carlo method simulation of the single photon Poisson distribution, and we took the root mean square difference of 200 simulation results.

Besides, we use the distribution of classical light intensity in space to estimate the state recovery in OAM space. In this case, we replace the single photon source by a classical laser. The intensity is observing with a Charge-Coupled Device (CCD) at a distance of approximately 2.8m. The light intensity is shown in Fig. S5. Though the distribution of light intensity in space only contains information about OAM state, it is helpful for estimate the decoherence in our QW platform. We calculate the deviation of the state before and after one cycle of walking in the OAM subspace, obtain the theoretical distribution, and compare it with experiments. We use probability similarity to estimate the role of decoherence in walking, as defined in the main text. Fig. S5 shows the recovery scenarios for three different initial states.

V. FULL STATE REVIVAL OF OTHER INITIAL STATES

Here we provide experimental information on the full state revival of state $\left|\psi_2^2\right\rangle$, which is the state from the initial state $\left|\psi_0^2\right\rangle$ evolution to time t=2. The state of $t = 2$ $\left|\psi_2^2\right\rangle$ and final state $\left|\psi_6^2\right\rangle$ reconstructed via quantum state

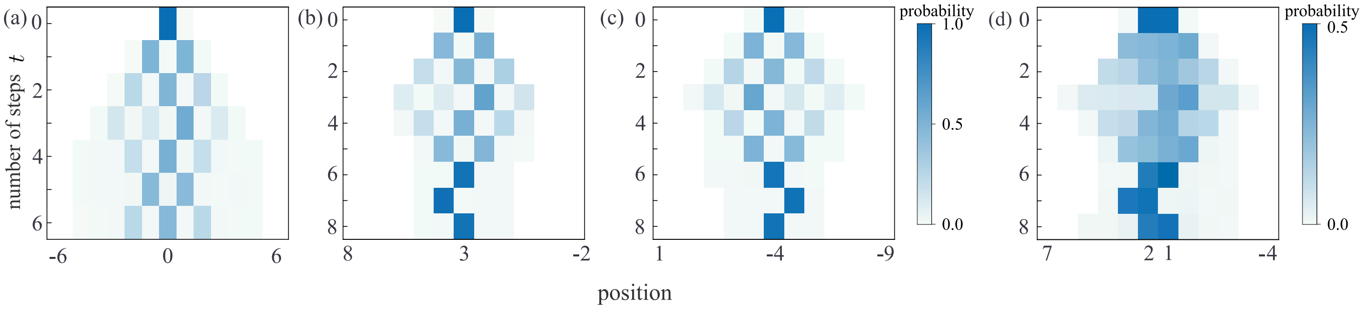

FIG. S7. (a) Measured probability distribution of the quantum walk with the initial state is $\left|\psi_0^2\right\rangle = |0\rangle \otimes |H\rangle$ and the coin flipping operator $C_1 : \phi_1 = \phi_2 = 0, \theta = \pi/4$. The state of the quantum walk after $t = 2$ steps is revived at $t = 6$, i.e., $\left|\psi_6^2\right\rangle = \left|\psi_2^2\right\rangle = \frac{1}{2}\left[-\left|-2\right\rangle \otimes |V\rangle + |0\rangle \otimes (|H\rangle + |V\rangle) + |2\rangle \otimes |H\rangle\right]$. (b-d) Experimental results of the probability distribution of the quantum walk with three different initial states $\left|\psi_0^4\right\rangle = |3\rangle \otimes |V\rangle$ (b), $\left|\psi_0^5\right\rangle = |-4\rangle \otimes |H\rangle$ (c), $\left|\psi_0^6\right\rangle = \frac{1}{\sqrt{2}}(|1\rangle + |2\rangle) \otimes |V\rangle$ (d), and the coin flipping operator $C_1 : \phi_1 = \phi_2 = 0, \theta = \pi/4$. The first intervention is introduced at $T = 4$.

tomography and we calculated the fidelity with the theoretical state $\left|\psi_2^2\right\rangle$(theoretical), $F_{t=2} = 0.8219 \pm 0.0003$ and $F_{t=6} = 0.7610 \pm 0.0017$. In addition, we provide probability distribution results show in Fig. S7. The results showed that we revival the state of $t = 2$ $\left|\psi_2^2\right\rangle$ through two intervention operations at $t = 6$.

We choose three initial states $\left|\psi_0^4\right\rangle = |3\rangle \otimes |V\rangle$, $\left|\psi_0^5\right\rangle = |-4\rangle \otimes |H\rangle$ and $\left|\psi_0^6\right\rangle = \frac{1}{\sqrt{2}}(|1\rangle + |2\rangle) \otimes |V\rangle$. Experimentally, the three initial states are prepared with the fidelities 0.9936 ± 0.0003, 0.9923 ± 0.0003, and 0.9481 ± 0.0013, respectively, which are reconstructed after 8-step evolution of the quantum walk through quantum state tomography in Fig. S1 (e,h-j). As shown in Fig. S7(b-d), the fidelities of the final states are 0.9491 ± 0.0006, 0.9449 ± 0.0011 and 0.8150 ± 0.0016, respectively, which show high reliability and generality of our experiment.

VI. ERROR ANALYSIS

In our experiment, the error sources include the efficiencies of the q plates, the spatial light modulator, and the avalanche photodiodes, respectively, and the accuracy of the wave plates. Among these, the efficiencies of the q plate (approximately 0.995) is the main source of the experimental imperfections. We have shown the numerical simulations by considering the efficiency of the q plates. As shown in Fig. S6, the efficiency of the q plates (approximately 0.995) leads to the experimental values being lower than the theoretical values.

VII. THE ADVANTAGES OF SINGLE PHOTONS

The coherent light can be used to simulate single photons. This is because for single-walker quantum walks, the probability distribution of single photons is similar to the intensity distribution of the simulated coherent light.

However, the measurement device for single photons is significant from that of coherent light. For example, our device for quantum walks of single photons can be directly applied to a local part of a multi-partite system, which cannot be simulated by coherent light when there are entanglements among these parties [3].

In this scenario of revival of unknown state, the potential advantages of two-photon or multi-photon sources are to implement revival of unknown two-photon or multi-photon entangled states, which can not be simulated with coherent light. Besides, for other scenarios of quantum walks, such as Boson sampling and random sampling, two-photon or multi-photon effect can also show advantages and novel phenomena compared to coherent light.

[1] Jayakody MN and Nanayakkara A et al. Reversion of quantum walks via interventions on coin space. *arXiv* 2018; **1803**: 08258.

[2] Jayakody MN, Paiva IL, Nanayakkara A et al. Induced on-demand revival in coined quantum walks on infinite d-dimensional lattices. *Phys Rev A* 2022; **105**: 032413.

[3] Jiao ZQ, Gao J, Zhou WH et al. Two-dimensional quantum walks of correlated photons. *Optica* 2021; **8**: 1129-1135.
